# “Essentials” and “Non-Essentials” in Dental Education

**Published:** 1918-10

**Authors:** Julio Endelman


### "ESSENTIALS" AND "NON-ESSENTIALS" IN DENTAL EDUCATION.

BY JULIO ENDELMAN, D.D.S.

It is an acknowledged fact in pedagogics that examinations at frequent intervals during the college year act as a stimulus for keener endeavor to both students and instructors. Such a system of repeated examinations as against the plan of one yearly examination at the end of the term possesses a number of valuable features and among which the one that has to do with relieving the student from the anxiety incident to the knowledge that his progress is to be gauged by the results of one single examination at the conclusion of the course is not to be overlooked. Mental anxiety is inimical to scholastic progress. Instructors imbued with a correct conception of teaching do not depend upon the finals as the sole means of gauging the student progress. A division of the college year into "quarters" with an examination at the end of each, in addition to remedying this possible mental pre-occupation, will help to impress the student with those features of the work previously covered which by his teachers are considered essential to his future success as a practitioner. The non-essential portions of the didactic work, and every subject taught is permeated with non-essentials, would unconsciously be relegated to a place of secondary importance. The division of the scholastic year into "quarters" is being adopted in a large number of collegiate, medical and dental institutions and thereby gains in time and efficiency are being accomplished. The medical and dental courses cover a multitude of subjects from which it is the duty of teachers to present to their classes those portions of the work which in their opinion are essential to a well rounded out dental and medical education. It is a fallacy to assume that four years or twice that number for that matter would be sufficient time in which to acquire *in toto* every subject taught in dental or medical schools. Such a feat would be way beyond the brain power of the average dental student and would probably add little to his assets as a dental practitioner. It is of far greater importance from the standpoint of professional efficiency to ground the student thoroughly in only such fundamentals as will enable him to perfect himself as a practitioner in the years following his graduation, rather than to encumber his mental processes with information which he will never be able to apply to the every-day duties of his specialty.

Dental educators must come to the inevitable conclusion, and some have already done so, that the imparting of information which can not be applied clinically, particularly in the teaching of the specialties, or which does not lead to a clearer understanding of the problems which practitioners in the specialty are bound to be confronted with is a pedagogic fallacy. It is up to the teachers in dental schools to separate the wheat from the chaff and to make the instruction in every branch of the curriculum utilitarian in its ultimate manifestation. The plan of quarterly examinations is of considerable value in that it does away with the necessity on the part of the student of weighting his brain throughout a long term with an amount of information which because it lacks in clinical application is easily forgotten. Once a quarterly examination is passed, those which are to follow should not contain questions upon the non-essentials previously covered and upon which the student has already been examined, but upon the "essentials" taught in the previous quarter or quarters and which students should retain indefinitely.

These innovations in dental education are aimed at greater efficiency by the elimination of antiquated time-wasting methods of teaching, for anything which is taught in dental schools which will not help the student to treat upon a scientific basis the diseases which come under the supervision of the dentist or which does not improve his technic adds not an iota to his professional assets.

The teaching of anatomy in dental schools is an instance of time-wasting methods of instruction. Recitations and lectures several times per week for one or two years, hundreds of precious hours spent in memorizing parrot-fashion the names of arteries, veins and nerves and of bony landmarks which have little or no bearing on operative surgery, and when graduation time comes the knowledge of *applied* anatomy of the majority of dental students is a pitiful exhibit of misdirected teaching. The same may be said of chemistry, metallurgy, physiology, comparative anatomy, etc.

Dental teachers are not in sympathy with time-wasting methods of teaching. They are willing and would be found ready to call the non-essentials from their courses, but in order to do so they require the collaboration of state dental boards.

Dental schools, when they accept students for innstruction, assume a solemn obligation to impart to them a well rounded out dental training, and in addition to this the information necessary to become legal practitioners in the state in which they are to locate, provided they have shown the proper degree of diligence throughout the course. As a consequence teachers do not feel warranted in eliminating from their courses any part or parts of them, for the boards may propound questions upon just such portions of the course considered non-essential by teachers. Co-operation must exist between dental teachers and board members if dental education is to be made more efficient by the elimination of the irrelevant and non-essential. There is not any more justification for teaching the non-essential parts of course than there is for dental boards to propound questions upon them.

